# Regression of left ventricular mass following conversion from conventional hemodialysis to thrice weekly in-centre nocturnal hemodialysis

**DOI:** 10.1186/1471-2369-13-3

**Published:** 2012-01-19

**Authors:** Ron Wald, Andrew T Yan, Jeffrey Perl, Depeng Jiang, M Sandra Donnelly, Howard Leong-Poi, Philip A McFarlane, Jordan J Weinstein, Marc B Goldstein

**Affiliations:** 1 Division of Nephrology, St. Michael's Hospital and the Univerity of Toronto, Toronto, Ontario, Canada; 2 Keenan Research Centre in the Li Ka Shing Knowledge Institute of St. Michael's Hospital, Toronto, Ontario, Canada; 3 Division of Cardiology, St. Michael's Hospital and the Univerity of Toronto, Toronto, Ontario, Canada; 4 Department of Community Health Sciences, University of Manitoba, Winnipeg, Manitoba, Canada; 5 Dalla Lana School of Public Health, University of Toronto, Toronto, Ontario, Canada

**Keywords:** end-stage renal disease, in-centre nocturnal hemodialysis, left ventricular mass

## Abstract

**Background:**

Increased left ventricular mass (LVM) is associated with adverse outcomes in patients receiving chronic hemodialysis. Among patients receiving conventional hemodialysis (CHD, 3×/week, 4 hrs/session), we evaluated whether dialysis intensification with in-centre nocturnal hemodialysis (INHD, 3×/week, 7-8 hrs/session in the dialysis unit) was associated with regression of LVM.

**Methods:**

We conducted a retrospective cohort study of CHD recipients who converted to INHD and received INHD for at least 6 months. LVM on the first echocardiogram performed at least 6 months post-conversion was compared to LVM pre-conversion. In a secondary analysis, we examined echocardiograms performed at least 12 months after starting INHD. The effect of conversion to INHD on LVM over time was also evaluated using a longitudinal analysis that incorporated all LVM data on patients with 2 or more echocardiograms.

**Results:**

Thirty-seven patients were eligible for the primary analysis. Mean age at conversion was 49 ± 12 yrs and 30% were women. Mean pre-conversion LVM was 219 ± 66 g and following conversion, LVM declined by 32 ± 58 g (p = 0.002). Among patients whose follow-up echocardiogram occurred at least 12 months following conversion, LVM declined by 40 ± 56 g (p = 0.0004). The rate of change of LVM decreased significantly from 0.4 g/yr before conversion, to -11.7 g/yr following conversion to INHD (p < 0.0001).

**Conclusion:**

Conversion to INHD is associated with a significant regression in LVM, which may portend a more favourable cardiovascular outcome. Our preliminary findings support the need for randomized controlled trials to definitively evaluate the cardiovascular effects of INHD.

## Background

Patients with chronic kidney disease (CKD) who require dialysis are at high risk of cardiovascular morbidity and mortality [1]. Interventions that have been shown to improve cardiovascular outcomes in the general population have inconsistently demonstrated benefit in patients receiving chronic dialysis [2-5]. This may be, in part, due to the unique array of cardiovascular risk factors in patients with end-stage renal disease (ESRD)[6]. Emerging data suggest that intensification of hemodialysis (HD) through the provision of more frequent and/or more prolonged sessions may reduce the high burden of cardiovascular disease in this population [7,8] and enhance survival [9,10]. Improved clinical outcomes may be mediated by reduction in the extracellular fluid volume and improved blood pressure control, enhanced clearance of uremic toxins, or more consistent mineral metabolic control [11-13].

While conventional HD (CHD) regimens are typically thrice weekly with each dialysis session lasting 3-4 hours, more intensified schedules have taken advantage of the nighttime for the administration of extended dialysis sessions, thereby avoiding intrusion into productive daytime hours. In a recent randomized controlled trial, home nocturnal HD (5-6 sessions per week, 7-8 hours per session) conferred a regression in left ventricular mass (LVM), a well-validated surrogate for clinical events, as compared to individuals who remained on CHD [7]. Unfortunately, only a minority of CHD patients is capable of undertaking home nocturnal HD and therefore these benefits of intensified HD may not be available to the majority of the conventional HD population.

In-centre nocturnal HD (INHD, 3 sessions per week, 7-8 hours per session) presents an alternative promising approach to intensified dialysis, particularly for individuals with barriers to self-administered home HD [14-19]. Although there is increasing interest in thrice weekly INHD, there are limited data on the cardiovascular impact of this modality. In this study, we evaluated the change in LVM, as measured by echocardiography, in relation to the conversion from CHD to INHD.

## Methods

This is a retrospective cross-over study of patients with ESRD who converted from CHD to INHD at St. Michael's Hospital, a tertiary care academic hospital in Toronto, Canada, from January 1, 2004 to September 30, 2009. The INHD Program at St. Michael's Hospital was initially instituted to accommodate chronic HD patients who might benefit from an intensified dialysis schedule but for whom there was a barrier to home dialysis. Individuals with hemodynamic instability during hemodialysis or poor mineral metabolic control on CHD in addition to those with compelling socioeconomic reasons (i.e., job preservation) for intensified nocturnal dialysis were preferentially referred for conversion to INHD. With increasing availability of INHD at our institution over time, patients receiving CHD who expressed a desire to switch to INHD for any reason were also considered. Patients who commenced INHD before October 1, 2009 and continued INHD for at least 6 months following conversion from CHD were eligible for inclusion in this study.

HD was administered in the CHD and INHD programs using the Gambro Phoenix (Gambro Inc, Richmond Hill, ON) HD machine and Baxter Exeltra 210 and Xenium 210 dialyzers which contain cellulose triacetate and polyethersulfone membranes, respectively (Baxter Healthcare Corp., Mc-Gaw Park IL). Patients who were on CHD received thrice weekly treatments of 3.5-4 hours duration with a target blood pump speed of 400 mL/min and dialysate flow rate of 500-800 mL/min. INHD was performed for 7-8 hrs thrice weekly with a dialysate flow rate of 500 mL/min and a target blood pump speed of 300 mL/min. In both modalities, anticoagulation was achieved with unfractionated heparin. A standard calcium bath of 1.25 mmol/L was used in CHD and was increased to 1.5 mmol/L when calcium-based binders were reduced or eliminated following conversion to INHD. When appropriate, diet was liberalized and phosphate binders tapered once INHD commenced. Patients were managed according to prevailing guidelines for blood pressure, hemoglobin, mineral metabolism parameters and vascular access [20-25].

The St. Michael's Hospital Research Ethics Board approved the study and due to the retrospective nature of the data collection, the requirement for informed consent was waived.

### Data collection

Clinical and demographic data were abstracted from the hospital's electronic information system and complemented by information from a dedicated database that tracks all patients in our HD program. For each laboratory value, the pre-conversion value was the mean of all readings obtained during the three months prior to INHD conversion. The post-conversion value comprised the mean of values obtained during the three months after the patient had been on INHD for 6 months and 12 months, respectively.

### Echocardiographic data

We used hospital databases to identify all transthoracic echocardiograms that were performed following the initiation of chronic hemodialysis. Pre- and post- conversion echocardiograms refer to the timing of the echocardiogram in relation to the date of the first INHD session. Echocardiographic data were systematically gleaned from reports by a trained data collector. LVM was the primary endpoint of this study; left ventricular mass index was not consistently available as contemporaneous patient weight and height were not recorded at the time of each echocardiographic examination.

Standardized echocardiographic examinations were performed, including 2-dimensional, M-mode and Doppler recordings. Exams were performed with high-quality commercially available echocardiographs equipped with 2.0 to 2.5 MHz transducers (IE33 Philips Ultrasound). Exams were digitally recorded (XCelera Echo, Philips Ultrasound) and reported off-line by highly-experienced readers, blinded to clinical data. LVM was calculated using an anatomically validated formula for echocardiography [26,27] and did not include the papillary muscles.

### Statistical analyses

In our primary analysis, we considered all patients with a pre-conversion echocardiogram at anytime between the initiation of chronic dialysis until conversion to INHD. These individuals were also required to have a post-conversion echocardiogram after at least 6 months of commencing INHD. A secondary analysis was restricted to a sub-cohort of individuals with a post-conversion echocardiogram that took place at least 12 months following the start of INHD. The paired t-test was used to evaluate the changes in LVM and laboratory measurements that were normally distributed. Laboratory measurements that were not normally distributed were compared with the Wilcoxon signed rank test.

The impact of conversion to INHD was assessed in an additional analysis by considering all available echocardiograms during each patient's dialysis history. Patients with at least 2 available echocardiograms at anytime were considered in these analyses. The number of echocardiograms ranged from 2 to 12 per patient. In analyzing such repeated measures data, individual differences in LVM were captured by random effects using growth modeling. The random effects represent continuous variation in growth from the patient's first available LVM and the change in LVM over time. In the within-patient component of mixed model analyses, a piece-wise linear model was used to estimate changes both before and after conversion to INHD. In the between-patient component of the analyses, we modeled the inter-individual differences in LVM [28,29].

Statistical analyses were performed using SAS Version 9 (SAS Institute Inc., Cary, North Carolina). All p-values were two-sided, and significance was set at a value of 0.05.

### Systematic re-evaluation of the echocardiographic data

The data used in our primary and secondary analyses were gleaned from reports of studies that were performed on clinical grounds in the context of usual care in our echocardiography laboratory. Since this approach may have introduced excessive variability into the estimation of LVM, a senior echocardiographer (H. L-P), performed a systematic review of all the echocardiograms for which pre- an post-INHD images were retrievable. The change in LVM following at least 6 months on INHD (which parallels the primary analysis above) was reported as a sensitivity analysis.

## Results

### Patient Characteristics

Fifty-six subjects initiated INHD between January 1, 2004 and September 30, 2009. Ten individuals were excluded as the required echocardiograms were not available. Nine people were excluded as they received less than 6 months of INHD before changing to another modality.

The characteristics of the 37 patients who were included in the primary analysis are shown in Table 1. Approximately 70% were men and the mean age was 49 ± 12 years. The median time since the start of renal replacement therapy was 4 years. The most common cause of ESRD was glomerulonephritis (35%) and this was followed by diabetes (32%). At the time of conversion to INHD, approximately half of the patients were dialyzed through an arteriovenous fistula. The majority of patients had preserved left ventricular ejection fraction prior to starting INHD.

**Table 1:** Description of patients converting to in-centre nocturnal dialysis (n = 37)

Female (%)	11 (30)
Age at time of conversion, in years	49.0 ± 12.2

Ethnicity (%)	
Caucasian	18 (49)
Black	4 (11)
Asian	3 (8)
Pacific Islander	8 (22)
Other	4 (11)

Etiology of ESRD (%)	
Diabetes mellitus	12 (32)
Ischemic	2 (5)
Glomerulonephritis	13 (35)
Other	4 (11)
Unknown	6 (16)

Median time on dialysis pre-conversion (IQR), in years	4.0 (1.4-8.3)

Vascular access at time of conversion	

Arteriovenous fistula	18 (49)
Arteriovenous graft	1 (2)
Central venous catheter	18 (49)

*Pre-INHD conversion echocardiographic data*	

Left ventricular ejection fraction *	
> 60%	26 (72)
40-59%	5 (14)
< 40%	5 (14)

LV end-diastolic dimension, in cm	4.9 ± 0.7
LV end-systolic dimension, in cm	3.3 ± 0.9
LV posterior wall thickness, in cm	1.2 ± 0.2
Interventricular septal wall thickness, in cm	1.2 ± 0.3
Patient weight, in kg	77.2 ± 19.9

Previous kidney transplant	13 (35)
Diabetes	17 (46)
History of coronary artery disease^#^	8 (22)
History of cerebrovascular disease	2 (5)
History of cancer	6 (16)

Categorical variables are expressed as number (%) and continuous variables are expressed as the mean ± standard deviation.

* Ejection fraction data missing for 1 patient

^# ^Coronary artery disease was defined as a history of myocardial infarction, coronary artery bypass graft or percutaneous coronary intervention.

### Changes in hematologic and biochemical parameters

The per-session urea reduction ratio increased by six months following the start of INHD and this was sustained at 12 months. There was a reduction in serum phosphate (1.57 ± 0.59 mmol/L at 6 months and 1.46 ± 0.47 mmol/L at 12 months post-conversion vs 2.10 ± 0.59 mmol/L pre-conversion, p < 0.0001 for both comparisons) and a rise in serum calcium (2.20 ± 0.12 mmol/L at 6 months post-conversion vs 2.12 ± 0.23 mmol/L pre-conversion, p = 0.03) following conversion to INHD (Table 2). Conversion to INHD was not associated with significant changes in intact parathyroid hormone, serum albumin or hemoglobin.

**Table 2:** Selected laboratory values prior to INHD conversion and 6 and 12 months following INHD conversion

	Pre-conversion (n = 37)	Six months following INHD conversion (n = 37)	Twelve months following INHD conversion (n = 31)
Reduction in urea, %	71.7 ± 6.7	84.9 ± 6.3*	86.7 ± 6.4*
Hemoglobin, in g/L	114.5 ± 17.3	119.1 ± 14.8	117.1 ± 13.5
Serum albumin, in g/L	33.7 ± 3.5	33.3 ± 3.0	32.8 ± 4.4
Calcium, in mmol/L	2.12 ± 0.23	2.20 ± 0.12*	2.18 ± 0.17
Phosphate, in mmol/L	2.10 ± 0.59	1.57 ± 0.49*	1.46 ± 0.47*
PTH, in pmol/L	26 (20-41)	28 (15-74)	36 (17-61)
ALP, in Units/L	95 (63-138)	124 (105-167)*	144 (92-212)*

Data are presented as means ± standard deviation or medians (interquartile range), as appropriate. The paired t-test was used to compare pre- and post-conversion differences for normally-distributed variables and the signed rank test was used for non-normally distributed variables.

* denotes p-value of < 0.05 for change in the laboratory value as compared to pre-conversion to INHD

### Left ventricular mass in relation to INHD conversion

In the primary analysis, a 32 ± 58 gram decline in LVM was observed following conversion to INHD (p = 0.0002) (Table 3). Some degree of LVM regression occurred in 29 of 37 (78%) patients. Among 31 patients who were eligible for the secondary analysis, which required a follow-up echocardiogram at least 12 months after conversion, the decline in LVM was greater (-40 ± 56 grams, p = 0.0004).

**Table 3:** Changes in left ventricular mass following conversion to INHD

Analysis*	Left ventricular mass (SD) in grams while on CHD	Left ventricular mass (SD) in grams after conversion to INHD	Change in LVM (SD) in grams	p-value
**Primary (n = 37)**	219 ± 66	186 ± 69	-32 ± 58	0.002

**Secondary (n = 31)**	215 ± 71	174 ± 62	-40 ± 56	0.0004

*In both the primary and secondary analyses, the patient's last available echocardiogram on conventional hemodialysis prior to conversion to INHD was considered the pre-conversion study. In the primary analysis, the post-conversion echocardiogram was the first study occurring ≥ 6 months following conversion to INHD. In the secondary analysis, the post-conversion echocardiogram was the first study occurring ≥ 12 months following conversion to INHD.

Our mixed model analysis included 184 studies from 40 patients who converted to INHD and had at least two echocardiograms performed at anytime during their dialysis history (Table 4). On average, before the conversion date, there was no significant change (slope = 0.4 ± 2.5 g/year, p = 0.87) in LVM. After the conversion date, there was a significant decrease in LVM (slope = -11.7 ± 3.1 g/year, p < 0.001). The annual change in LVM following conversion differed significantly from that before the conversion date (-12.1 ± 7.8 g/year, p = 0.012).

**Table 4:** Effect of INHD Conversion on Left Ventricular Mass

	Estimate (Standard Error)
Level of LVM at the conversion date (g)	212.9 (9.9) *
Annual change in LVM before the INHD conversion date (g/year)	0.4 (2.5)
Annual change in LVM following the INHD conversion date (g/year)	-11.7 (3.1)*
Difference in annual change in LVM following and prior to INHD conversion (g/year)	-12.1(4.8) *

The results are based on estimates from a mixed model analysis. They include all patients who converted to INHD who had at least 2 echocardiograms performed at anytime (n = 40). All available echocardiograms for these patients were considered in these analyses (n = 184).

* = p < .05

### Sensitivity analysis

We were able to retrieve the electronic images of pre- and post-INHD conversion echocardiographic images in 17 individuals included in the primary analysis and these were read by a single blinded echocardiographer. As compared to values obtained before conversion to INHD, the mean reduction in LVM obtained at least 6 months following INHD conversion was 31 ± 43 grams (p = 0.001).

## Discussion

We have previously shown that INHD is a feasible and well-accepted form of renal replacement therapy for patients with ESRD [14]. Our current study expands on these findings by demonstrating that conversion to INHD is associated with a significant regression in LVM.

Left ventricular hypertrophy (LVH) is common in patients receiving chronic dialysis and is likely the consequence of many factors that are prevalent in patients with chronic kidney disease, including longstanding hypertension, extracellular fluid volume expansion, endothelial dysfunction, inflammation, oxidative stress and altered mineral metabolism [30-32]. In the general population as well as in those with ESRD, LVH is associated with higher rates of cardiovascular events including sudden death and arrhythmias [32]. Regression of LVM has been associated with improved outcomes in patients with and without ESRD [33-35].

There is a growing body of data suggesting that intensified dialysis, as delivered through home nocturnal HD 5-6 times per week [7,36], is associated with LVM regression. We hypothesized that these benefits may extend to individuals receiving thrice weekly INHD. A recently published cohort study suggested lower mortality and a 24 g/m^2 ^reduction in left ventricular mass index after one year of INHD [18]. However, only about one-third of patients underwent both baseline and follow-up echocardiography. In the current study, we confirmed the association between conversion to INHD and LVM regression after analyzing pre- and post-INHD echocardiograms in the majority of our patients who commenced INHD.

Despite the fact that INHD delivers less than half of the total dialysis time administered through home nocturnal regimens, the magnitude in LVM regression that we observed was comparable to that achieved in studies that demonstrated the benefits of home nocturnal hemodialysis [7,36]. In a cohort study of 28 patients who commenced home nocturnal hemodialysis, Chan et al showed a 17 g/m^2 ^reduction in indexed LVM at one year [36]. A clinical trial in Alberta, Canada, demonstrated a 14 g reduction in LVM after 6 months of nocturnal hemodialysis [7]. The recently reported Frequent Hemodialysis Network trial demonstrated a non-significant reduction in LVM of 8 g after 1 year of home nocturnal hemodialysis [37]. The more modest findings in the latter trial may be related to the inclusion of recipients with a shorter dialysis vintage and greater residual kidney function. Overall, the advent of INHD may represent an important means of providing intensified dialysis to the vast majority of ESRD patients who would not be capable or would be otherwise unwilling to adopt home HD [38]. Although the cardiovascular benefits of intensified dialysis are likely maximized by self-administration of therapy on most nights of the week, three sessions per-week allows us to provide therapy to more patients.

This study has several limitations. The commencement of INHD was often dictated by clinical (eg, severe hyperphosphatemia, hemodynamic lability on conventional dialysis) or socioeconomic (eg, job preservation) circumstances. As a result, applicability of our findings to the broader hemodialysis population is not assured. Since this was a retrospective review, the echocardiographic data emanate from studies which were not carried out at regular pre-defined intervals. Some INHD recipients lacked pre- and/or post-conversion echocardiograms and could not be studied in our main analyses while other patients had numerous studies, likely driven by clinical circumstances. These factors may limit the generalizability of our findings. We aimed to address these factors by performing an analysis that accounted for all echocardiograms in each individual with at least two available studies. Using mixed models, we were able to account for variable time intervals between studies. Reassuringly, the significant post-INHD reduction in LVM persisted when using this analytic approach.

We did not consider a control group of patients who remained on CHD. While recent studies that evaluated intensified dialysis attempted to identify matched controls [10,19], such matching is often difficult as patients who agree to intensify their dialysis regimen may be inherently different than those who do not. Allowing patients to serve as their own controls using a cross-over design mitigates against confounding by patient-specific characteristics that are often difficult to measure. Importantly, recent work has shown that LVM generally increases with time in incident patients who receive CHD [39]. In our patients who converted to INHD, all aspects of routine dialysis care did not fundamentally change over time. This suggests that the echocardiographic changes that we observed are likely the result of dialysis intensification with INHD.

LVM estimated by echocardiographic methods has limited precision and is susceptible to rapid changes in extracellular fluid volume that occur during dialysis [40]. The timing of the echocardiogram with respect to dialysis was not standardized thereby causing inevitable variability in the LVM estimates. Furthermore, some echocardiograms may have been obtained for specific clinical reasons, which could impact on the results we obtained. While INHD provides more intensive dialysis, the interdialytic interval remains essentially unchanged from CHD, and thus interdialytic weight gain is less likely to change following conversion to INHD. Moreover, the random relationship between the timing of echocardiography and dialysis was present both before and after conversion to INHD. As a result, there should be no systematic bias that would impact on LVM estimates in either phase of the study. In our main analyses, echocardiograms were obtained over a timeframe of several years and were interpreted by multiple readers. This may have contributed to variability in the readings. Importantly, a sensitivity analysis whereby a single senior echocardiographer reviewed all retrievable studies confirmed a LVM reduction that was consistent with the results obtained in the primary analysis.

Cardiac magnetic resonance provides accurate and precise measurements of left ventricular structure [41,42] and has been repeatedly employed as the metric in trials of intensified dialysis [7,8,37,43]. Our group has adopted cardiac magnetic resonance-derived LVM as the primary outcome in an ongoing prospective evaluation of the cardiovascular effects on INHD (ClinicalTrials.gov registration NCT00718848). However, echocardiography is logistically easier to perform, has no contraindications, is far less expensive and remains the most widely used modality for the assessment of cardiac structure and function in clinical practice.

Irrespective of imaging technique, LVM regression is still a surrogate outcome. Left ventricular size may merely be reflective of the accumulated cardiovascular toxicity of end-stage renal disease and regression of LVM may indicate an attenuation of these effects. However, this does not imply that improvements in LVM are causally associated with better clinical outcomes. A well-designed randomized controlled trial that is based on clinically-relevant endpoints (i.e., all-cause mortality and adjudicated cardiovascular events) is required to provide clinical meaning to our findings and help determine whether the widespread adoption of intensified dialysis modalities such as INHD is justified. To our knowledge, there are no ongoing randomized trials exploring the impact of INHD on hard clinical endpoints. Until such studies are completed, observational studies using well-validated surrogate endpoints such as LVM regression provide valuable insights.

Finally, this study can not draw definitive conclusions on the mechanisms through which INHD may mediate LVM regression. Blood pressure lowering likely plays a key role in the LVM regression seen following the initiation of home nocturnal hemodialysis [7,36]. Others have shown that INHD may also lead to a reduction in blood pressure and/or the requirement for blood pressure-lowering medications [18,19]. Unfortunately, the retrospective nature of this study precluded the longitudinal analysis of blood pressure values and antihypertensive medication burden in relation to serial changes in LVM. Other factors that may impact on changes in LV mass, but which we were unable to capture, include dialysate composition, dialysis adequacy (as defined by standard Kt/V_urea_), residual kidney function, interdialytic weight gain and vascular access. Consideration of these factors will be important in future studies examining the cardiovascular impact of INHD.

## Conclusions

Our preliminary findings extend previous work and raise the possibility that the intensification of hemodialysis with thrice weekly in-centre nocturnal hemodialysis leads to a regression in left ventricular mass. If these findings are confirmed by more rigorous prospective studies, this may prompt a re-evaluation of the optimal way in which hemodialysis should be delivered.

## Competing interests relevant to this article

The authors declare that they have no competing interests.

## Authors' contributions

RW conceived the study, collected and analyzed the data, and wrote the manuscript. AY conceived the study, analyzed the data, and co-wrote the manuscript. JP conceived the study, analyzed the data, and co-wrote the manuscript. DJ helped design the study, performed the advanced statistical analyses and reviewed the manuscript. SD helped conceive the study and provided critical review of the manuscript. HLP helped conceive the study, collected echocardiographic data and provided critical review of the manuscript. PM helped conceive the study and provided critical review of the manuscript. JJW helped conceive the study and provided critical review of the manuscript. MBG conceived the study, collected and analyzed the data, interpreted the data and provided critical input to preparation of the manuscript. All authors read and approved the final version of the manuscript.

## Pre-publication history

The pre-publication history for this paper can be accessed here:

http://www.biomedcentral.com/1471-2369/13/3/prepub
